# A cross-sectional study: a breathomics based pulmonary tuberculosis detection method

**DOI:** 10.1186/s12879-023-08112-3

**Published:** 2023-03-10

**Authors:** Liang Fu, Lei Wang, Haibo Wang, Min Yang, Qianting Yang, Yi Lin, Shanyi Guan, Yongcong Deng, Lei Liu, Qingyun Li, Mengqi He, Peize Zhang, Haibin Chen, Guofang Deng

**Affiliations:** 1 Division Two of the Pulmonary Diseases Department, The Third People’s Hospital of Shenzhen, National Clinical Research Center for Infectious Disease, Southern University of Science and Technology, Shenzhen, 518112 China; 2 Breax Laboratory, PCAB Research Center of Breath and Metabolism, Beijing, 100074 China; 3 Peking University Clinical Research Institute, Peking University First Hospital, Beijing, 100000 China; 4 Institute for Hepatology, The Third People’s Hospital of Shenzhen, National Clinical Research Center for Infectious Disease, Southern University of Science and Technology, Shenzhen, 518112 China; 5 Medical Examination Department, The Third People’s Hospital of Shenzhen, National Clinical Research Center for Infectious Disease, Southern University of Science and Technology, Shenzhen, 518112 China; 6 Pulmonary Diseases Out-Patient Department, The Third People’s Hospital of Shenzhen, National Clinical Research Center for Infectious Disease, Southern University of Science and Technology, Shenzhen, 518112 China

**Keywords:** Pulmonary tuberculosis, Machine learning, Volatile organic compounds, Breathomics

## Abstract

**Background:**

Diagnostics for pulmonary tuberculosis (PTB) are usually inaccurate, expensive, or complicated. The breathomics-based method may be an attractive option for fast and noninvasive PTB detection.

**Method:**

Exhaled breath samples were collected from 518 PTB patients and 887 controls and tested on the real-time high-pressure photon ionization time-of-flight mass spectrometer. Machine learning algorithms were employed for breathomics analysis and PTB detection mode, whose performance was evaluated in 430 blinded clinical patients.

**Results:**

The breathomics-based PTB detection model achieved an accuracy of 92.6%, a sensitivity of 91.7%, a specificity of 93.0%, and an AUC of 0.975 in the blinded test set (n = 430). Age, sex, and anti-tuberculosis treatment does not significantly impact PTB detection performance. In distinguishing PTB from other pulmonary diseases (n = 182), the VOC modes also achieve good performance with an accuracy of 91.2%, a sensitivity of 91.7%, a specificity of 88.0%, and an AUC of 0.961.

**Conclusions:**

The simple and noninvasive breathomics-based PTB detection method was demonstrated with high sensitivity and specificity, potentially valuable for clinical PTB screening and diagnosis.

## Key messages

- What is already known on this topic—Breath VOC analysis is a potential technology for PTB detection. However, it is still desirable for a real-time, robust, accurate, and simple breath analysis platform for clinical application.
- What this study adds—An online breath detection for PTB was proposed and demonstrated with high sensitivity and specificity in a large clinical cohort.
- How this study might affect research, practice, or policy—This study may promote the application of breath detection in clinical TB detection and related biomarker studies.

## Introduction

Tuberculosis (TB) continues to be a major global health threat, with an estimated 10 million incident cases and 1.4 million deaths per year globally. In 2019, only 57% of pulmonary TB cases were confirmed by bacteriological examination. There is still a large gap, 2.9 million cases in 2019, between reported and estimated cases [1]. The absence of available technology for the timely and accurate detection of TB has been one of the major impediments to preventing and ending TB. Undiagnosed TB is associated with substantial morbidity and mortality and leads to ongoing TB transmission in the community, which makes improving the performance and delivery of diagnostic testing services a leading priority [2].

Sputum-based TB diagnostics are usually either inaccurate, expensive, or complicated in their usage [3]. Sputum specimens are difficult to collect, process, and transport, and only one-third of suspected TB patients can give adequate high-quality sputum samples [4], while it is even harder in children, HIV-infected patients, and those with extrapulmonary TB. Acid-fast bacilli staining of sputum has a high false-negative rate (up to 50%) [5]. The culture of sputum alone has a poor sensitivity of approximately 30% [6, 7]. GeneXpert MTB/RIF (Xpert) achieved good performances in TB detection and drug resistance testing in the clinic and has been recommended by the WHO. However, it still requires good infrastructure and sputum samples [8, 9]. WHO has identified four high-priority test types for diagnostic development and created target product profiles (TPPs) for each, among which some non-sputum tests should be offered [10]. Thus, there is a greater need than ever for fast, accurate, and non-sputum TB detection technologies.

Breathomics, a branch of metabolomics, is a promising tool because of its significant advantages: good accessibility, noninvasiveness, and specificness [11, 12]. A breath test could diagnose TB by detecting volatile organic compounds (VOCs) produced by mycobacterium tuberculosis (*M.tb*) and the infected host, which has been approved by many studies [13]. The most commonly used breath detection methods for TB diagnosis include gas chromatography–mass spectrometry (GC–MS) [14, 15] and electric or chemical sensors [16]. For GC–MS based studies, Phillips et al. used GC–MS to detect the VOCs in the exhalation of pulmonary TB (PTB) patients with positive culture results and healthy controls (HC), and the headspace air of M.tb culture flask. They found that patients' expiratory VOCs were similar to culture VOCs in naphthalene, 1-methyl- and cyclohexane, 1,4-dimethyl-. Based on the small sample modeling on 12 identified VOCs, the author obtained a sensitivity of 82.6% and specificity of 100%, which verified the feasibility of the breath test for PTB detection [17]. They further validated the VOCs-based PTB detection method within a larger transcontinental and ethnic group of 226 symptomatic high-risk patients in United States, Philippines, and United Kingdom, which achieves an overall accuracy of approximately 85% [18]. Beccaria et al. also used GC–MS to analyze the VOCs of exhaled breath of patients with active PTB and health controls in South Africa, achieving a sensitivity of 100% and specificity of 60% via the random forest method [19]. In addition, they performed another validation study using two-dimensional GC–MS for breath analysis on PTB and PTB-free patients in Haiti and found that a random forest model based on 22 characteristics VOCs can distinguish well between PTB and PTB-free patients, in which 2-butyl-1-octanol was the most expressed in the breath of TB positive population and was detected in 85% of this group (12/14), while only in 50% in the control group (10/20) [20]. 2-butyl-1-octanol was also identified by fuzzy logic analysis as the best discriminator between patients whose sputum cultures were positive or negative for Mycobacteria in Phillips’s study [17]. Bobak et al. conducted an exploratory study on the exhaled diagnosis of PTB in 31 children in South Africa and found that PTB could be identified with 90% accuracy from other respiratory infections based on four VOCs, including decane and 4-methyloctane[21]. Furthermore, the sensor based breath test method also achieved good performance on TB/PTB detection. For example, Marcel et al. constructed and evaluated a DiagNose (C-it BV) based TB diagnosis method on 194 participants, and achieved a sensitivity of 93.5% and a specificity of 85.3% in discriminating TB patients and HC, and got a sensitivity of 76.5% and specificity of 87.2% when identifying TB patient within the entire test-population [22]. Morad et al. evaluated a nano-sensor based TB detection method on 60 blinded validation datasets, and achieved a specificity, positive predictive value (PPV), and negative predictive value (NPV) of 88%, 76%, and 94%, respectively [23]. In 2017, Mohamed et al. distinguished TB patients (260) from HC participants (240) for multiple biological samples (blood, breath, sputum, and urine) with sensitive and specificity > 95% via e-Nose analyses [24]. The above studies proved the feasibility of breath VOCs based PTB detection.

GC–MS has advantages in the qualitative and quantitative detection of substances. However, the selection of chromatography columns and the complex procedures limited the detection scope of GC–MS. Besides, the consistency of reported VOCs from different studies is poor, since GC–MS analysis requires complex procedures and specialized skills [13]. The sensor based solution usually uses a single or a series of sensor to identify the response pattern to breath without considering the specific compositions. It is fast but easily affected by other interference factors such as the environment [13]. Thus, it is still desirable for a real-time, robust, accurate, and simple breath analysis platform for VOC detection. The online mass spectrometry platform could meet such requirements.

Recently, different online mass spectrometry technologies have been developed to analyze exhaled breath, such as proton transfer reaction MS (PTR-MS) [25], secondary electrospray ionization MS (SESI-MS) [26, 27], and high-pressure photon ionization time-of-flight mass spectrometry (HPPI-TOF-MS) [28]. The HPPI-TOFMS platform is designed and developed by our team and has been used for lung cancer and esophageal cancer detection [29–31] and achieved good performances with sensitivity and specificity > 90%. In this study, we aimed to develop a breathomics based PTB detection and investigate its performance on the clinical data set in this study.

## Methods

### Study design and participants

We conducted a cross-sectional study from 1 March 2020 to 31 March 2021 at The Third People's Hospital of Shenzhen. The study was approved by the Ethics Committee of The Third People's Hospital of Shenzhen (number: 2020-012). Written informed consent was obtained from all participants.

**Fig. 1:**
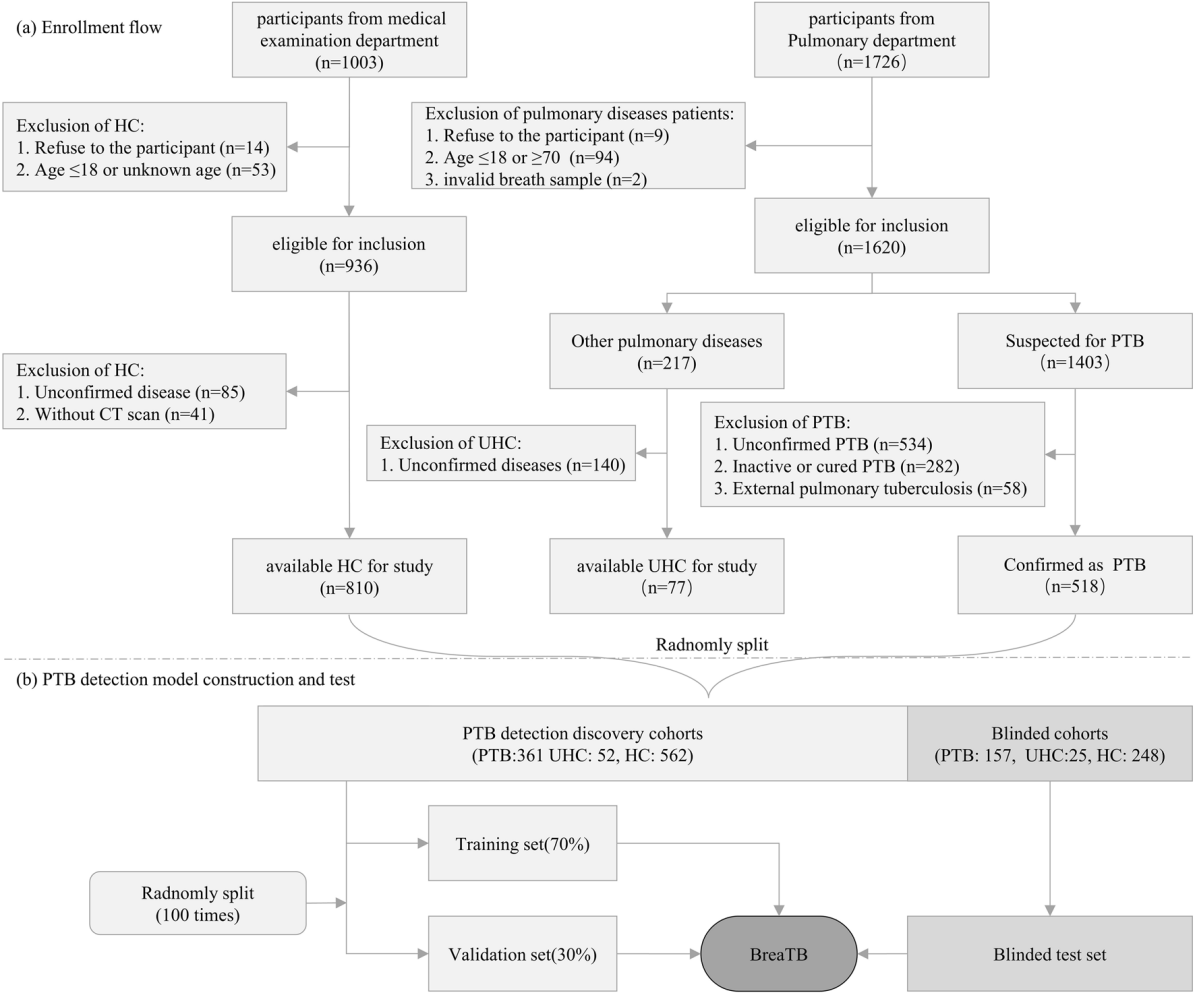
The flow of participants enrollment and PTB detection model construction and test

The total participants consisted of a case group and a control group. For the case group, confirmed PTB patients were prospectively and consecutively recruited based on the following criteria: (1) aged 18–70 years old; (2) diagnosed by Xpert and/or culture, with suggestive clinical and radiological findings; (3) anti-TB treatment not initiated or started less than 2 weeks. The control group consists of two parts: healthy controls with no pulmonary diseases (HC) and patients with pulmonary diseases (unhealthy controls, UHC) which could be noninfectious diseases or infectious diseases other than PTB. HCs were simultaneously recruited and underwent a physical examination with the following criteria: (1) aged 18–70 years old; (2) no respiratory symptoms (e.g., cough, sputum, hemoptysis, shortness of breath, dyspnea, or chest pain); (3) no pulmonary lesions by chest imaging (chest X-ray or computed tomography). For UHC, they should: (1) aged 18–70 years old; (2) have pathogenic confirmed infectious diseases or treatment response suggestive of pulmonary infectious diseases, or have chronic noninfectious diseases, without evidence of infection. Both the case group and the control group would be excluded if the airbag leaked or were unable to take enough breath volume. The participant enrollment flow is illustrated in Fig. 1a. A total of 518 PTB patients and 887 controls with 77 UHC and 810 HC were enrolled in this study.

The physicians were responsible for making a clinical diagnosis and for the collection of the breath samples. The other researchers performed the VOCs detection and ML modeling and were blinded to clinical data and other test results. Additionally, the physicians were also blinded to the breath test results. The demographic and clinical characteristics of all participants were collected and summarized in Table 1, including age, sex, and antituberculosis therapy.

**Table 1:** Demographic characteristics of participants

	Discovery data set			Test data set		
	PTB (N = 361)	Control (N = 614)	p-value	PTB (N = 157)	Control (N = 273)	p-value
Age						
Median (min.–max.)	36 (18–70)	28 (18–69)	** < 0.001**	32 (18–70)	28 (18–70)	** < 0.001**
< 30 (%)	115 (31.9)	345 (56.2)	**0.008**	64 (40.8)	169 (61.9)	0.258
≥ 30 (%)	246 (68.1)	269 (43.8)	** < 0.001**	93 (59.2)	104 (38.1)	**0.009**
Sex						
Male (%)	223 (61.8)	325 (52.9)	**0.009**	101 (64.3)	142 (52.0)	**0.004**
Female (%)	138 (38.2)	289 (47.1)	–	56 (35.7)	131 (48.0)	–

Bold p-value shows that there are significant differences between PTB and controls

### Sampling procedures

All breath samples were collected using a predefined protocol and tested within twenty-four hours. The sampling apparatus was composed of a disposable gas nipple and a sampling bag made of polyether-ether-ketone (PEEK). In this study, we set standard sampling demands and protocols to minimize the influence of the daily diet. Firstly, we conducted sampling at a second visit if he/she was an inpatient and informed the participants to prepare for sampling in advance: no smoking, alcohol, or diets within an hour before sampling. Secondly, participants were required to rinse their mouths with purified water instantly before sampling to minimize the influence of diet, smoking, etc. Thirdly, all samples are required to be collected in the same environment, which could minimize the effects of environmental facts. With a deep nasal inhalation, participants completely exhaled the air into the sampling bag with over 1.2 L volume.

### Breath sample detection

HPPI-TOFMS, which consisted of a vacuum ultraviolet (VUV) lamp-based HPPI ion source and an orthogonal acceleration time-of-flight (TOF) mass analyzer, was used to detect and analyze the breath samples. A commercial VUV-Kr lamp with a photon energy of 10.6 eV was adopted in this platform. Most VOCs with an ionization potential lower than 10.6 eV were ionized in the ionization region directly [32]. Breath samples were directly introduced through a 250 μm i.d. 0.60 m long stainless-steel capillary. The HPPI ion source works in soft HPPI ionization mode, which will produce mostly radical cations (M^+^) by ionization reaction as M + hγ → M^+^  + e. Then, the ion transmission system effectively transferred these ions from the ion source into the orthogonal acceleration, reflection TOFMS mass analyzer. The TOFMS signals were recorded by a 400 ps time-to-digital conversion rate at 25 kHz, and all the mass spectra were accumulated for 60 s. Thus, it takes 1 min for one sample to go through a detection. A spectrogram with 31,666 data pairs was extracted from each exhaled breath sample. Based on the flight time and m/z calibration on the standard gas with nine compounds at a concentration of 1 ppmv, the timeline of flight can be transferred as m/z, which is in the range of (0, 350). The TOFMS signals were positively correlated with the concentration of the VOC ions. The detection limit is down to 0.015 ppbv (parts per billion by volume) for aliphatic and aromatic hydrocarbons [28]. The gas-phase breath sample was directly inhaled into the ionization region through a 250 μm i.d. 0.60 m long capillary from the sampling bag. The TOF signals were recorded by a time-to-digital converter, and all the mass spectra were accumulated for 60 s. Mass spectrum peaks with m/z < 350 were detected by HPPI-TOFMS for each exhaled breath sample. The noise-reducing and base-line correction were implemented via anti-symmetric wavelet transformation, which was achieved by Python package pywavelets [33]. To transfer the discrete signal of mass spectra data to standard breathomics data, we calculate the area of the strongest peak in the range of [x − 0.1, x + 0.1) as the feature of VOC with m/z close to x. In this study, 1500 breathomics data were detected for machine learning (ML) model construction in the ions m/z range of [20, 320) with an interval of 0.2. A statistical analysis based feature selection was executed to avoid model over-fitting, in which the features without significant difference (p > 0.05) were excluded before model training.

### PTB detection model construction

As illustrated in Fig. 1b, all the enrolled participants were randomly split into two groups: 70% of them for model construction and the remaining 30% of them for model blinded testing. Thus, 361 PTB patients and 614 controls were randomly selected as the discovery data set. Through 100 times of 7:3 randomization, the discovery data set was further divided into a training subset and an internal validation subset. On the training subset, several popular ML models including Random Forest (RF) [34], Support Vector Machine (SVM) [35], Logistic Regression (LR) [36], eXtreme Gradient Boosting (XGB) [37], and Decision Tree (DT) [38] were employed as the classifier to distinguish PTB patients and controls. The descriptions and main parameter settings of these ML models are illustrated in Table 2. Then, the optimal classifier for distinguishing PTB patients and controls is selected according to the model performance in the internal validation subset, which is named as “BreaTB”.

**Table 2:** The descriptions and main parameter settings of the employed ML models

ML models	Descriptions	Main parameter settings^a^
RF	A meta estimator that fits a number of decision tree classifiers on various sub-samples of the dataset and uses averaging to improve the predictive accuracy and control over-fitting	n_estimators = 100, max_features = 0.5, min_samples_split = 4, min_samples_leaf = 10, criterion = "entropy"
SVM	Solves the separation hyperplane which can divide the training data set correctly and has the maximum geometric interval	penalty = "l2", loss = "squared_hinge", tol = 1e−5, C = 5.0, max_iter = 1e + 5
LR	Estimates the probability of an event occurring based on a given dataset of independent variables	tol = 1e−5, C = 5.0, max_iter = 1e + 4
XGB	A boosting algorithm based on gradient boosted decision trees algorithm	booster: "gbtree", max_depth: 8, n_estimators: 100, min_child_weight: 3, gamma: 0.15, lambda: 2
DT	Employs a divide and conquer strategy by conducting a greedy search to identify the optimal split points within a tree	criterion = "gini", splitter = "best", min_samples_split = 2, min_samples_leaf = 1

^a^These algorithms were achieved based on python packages: xgboost (https://xgboost.readthedocs.io/en/stable/python/python_intro.html) and sklearn (https://scikit-learn.org/stable/user_guide.html)

### Performance evaluation and statistical analysis

As BreaTB is constructed, the most important features can be confirmed based on the feature importance or coefficient in model training. Feature differences analysis was also implemented on the relative density of VOCs among different patient groups.

BreaTB was applied and evaluated on the blinded testing data set, which consisted of 157 PTB patients, 248 HC, and 25 UHC. The model detection results were compared with the clinically confirmed diagnosis results. Furthermore, we also assessed the performance of BreaTB stratified by clinical characteristics. We calculated the sensitivity, specificity, PPV, NPV, accuracy, AUC (the area under the receiver operating characteristic curve (ROC)), and the relative 95% confidence interval (CI) were calculated to evaluate the performance of BreaTB.

All statistical analyses were performed using SAS version 9.4 (SAS Institute Inc., Cary, NC, USA) and Origin software (version 2018). Descriptive statistics were reported as frequencies (percentages) for categorical variables or median (minima to maxima) for continuous variables. We compared the demographic characteristics among different patient groups using the Mann–Whitney U test for continuous variables and the chi-square test for categorical variables. A p-value < 0.05 was considered statistically significant in all analyses. All the tests were two-tailed.

## Results

**Fig. 2:**
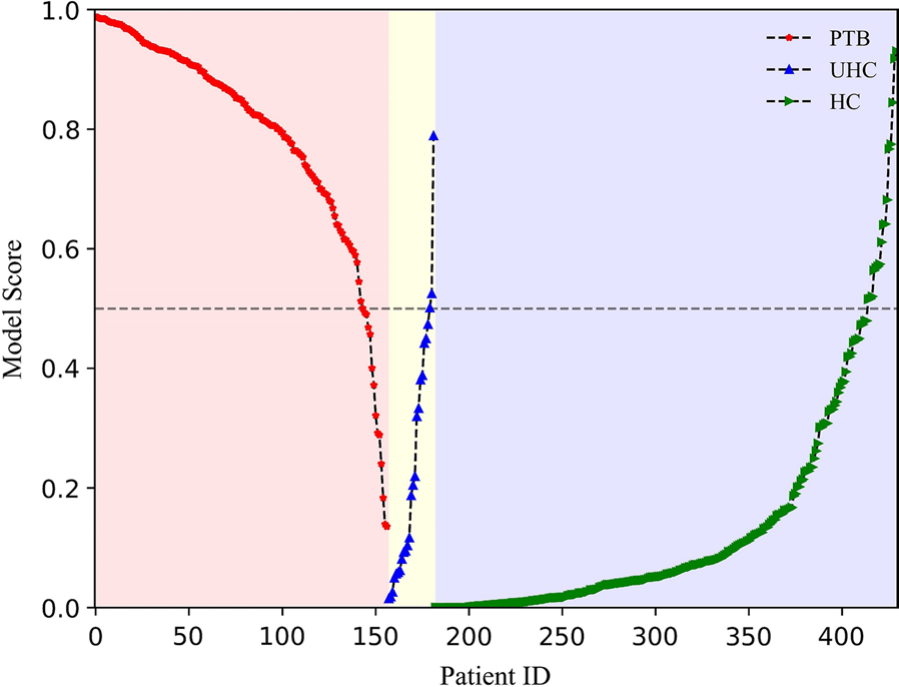
Predictive score of BreaTB on the test data set

For different ML models, the mean performance metrics of 100 models on randomly selected training sets were illustrated in Table 3. Since the scale of the dataset enrolled is relatively large in this study, these basic classifiers such as SVM, LR, and DT all perform well in the PTB detection task. As the meta and boosting classifiers of DT, the RF and XGB based PTB detection models have superior performances. Based on the validation results, the best-performing RF and XGB based PTB detection models were selected for further testing. The results in Table 3 showed the XGB model has better performance than the RF model in the validation data set. However, the RF model performs superior to the XGB model in the blinded test data set with an accuracy of 92.6% (95% CI 90.1–95.0%), a sensitivity of 91.7% (95% CI 88.5–95.0%), and a specificity of 93.0% (95% CI 88.9–97.2%). It implies that the RF model is more robust than XGB. Thus, we only further analyze the RF-based PTB detection model (termed as BreaTB). Figure 2 illustrated the prediction scores of BreaTB on all tested samples, which represent the probability of PTB infection. The cut-off line(threshold = 0.5) divides the PTB patients from controls well with fewer false positives and false negatives.

**Table 3:** Performance metrics (mean ± STD) of difference ML models for PTB detection in internal validation and blinded test dataset

Data sets	Models	Sensitivity (%)	Specificity (%)	PPV (%)	NPV (%)	Accuracy (%)	AUC
Validation (n = 295)	**RF**	**90.6 ± 3.1**	90.6 ± 2.4	85.1 ± 3.2	**94.3 ± 1.7**	90.6 ± 1.7	0.960 ± 0.011
	SVM	67.7 ± 20.0	83.4 ± 12.6	74.3 ± 10.4	83.1 ± 7.6	77.6 ± 4.8	0.755 ± 0.061
	LR	78.6 ± 4.4	82.0 ± 4.1	72.2 ± 4.7	86.8 ± 2.5	80.8 ± 3.1	0.856 ± 0.030
	**XGB**	88.1 ± 3.0	**93.6 ± 2.1**	**89.0 ± 3.2**	93.1 ± 1.6	**91.5 ± 1.6**	**0.969 ± 0.010**
	DT	76.1 ± 5.0	90.5 ± 2.8	82.6 ± 4.2	86.7 ± 2.4	85.2 ± 2.5	0.833 ± 0.028
Test (n = 430)	**RF**	**90.7 ± 1.5**	92.1 ± 1.5	86.9 ± 2.1	**94.5 ± 0.8**	91.6 ± 1.0	0.970 ± 0.005
	SVM	69.4 ± 20.4	83.6 ± 12.7	74.5 ± 9.7	84.3 ± 7.8	78.4 ± 5.2	0.765 ± 0.066
	LR	82.5 ± 3.3	83.2 ± 4.0	74.1 ± 4.5	89.2 ± 1.8	82.9 ± 2.8	0.877 ± 0.021
	**XGB**	88.1 ± 1.7	**94.6 ± 1.2**	**90.5 ± 2.0**	93.2 ± 0.9	**92.2 ± 0.9**	**0.970 ± 0.004**
	DT	75.3 ± 4.1	89.4 ± 1.9	80.5 ± 3.0	86.3 ± 2.0	84.3 ± 1.9	0.824 ± 0.023

Bold values represent the best performance metrics achieved among differences mahcine learning methods

**Fig. 3:**
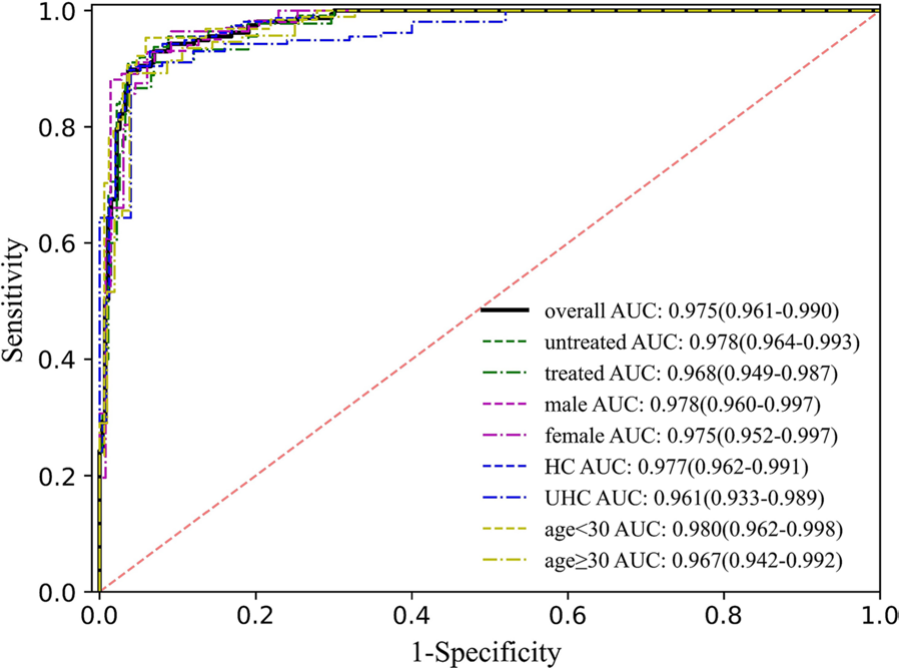
Performance of the BreaTB on different tuberculosis subgroups

As shown in Table 1, in the training data set, the median age of PTB patients was significantly higher than that of controls (36 (18–70) vs. 28 (18–69) years old), and there were more males in PTB patients than in controls (61.8% vs. 52.9%). The distribution of age ≥ 30 and gender in the test data set is as same as that in the training data set, except for that in age < 30. Thus, it is necessary to evaluate the influence of these clinic characteristics on model performance. As illustrated in Fig. 3 and Table 4, the ROC curve showed that BreaTB achieved an AUC of 0.975 (95% CI, 0.961–0.998) in the overall test data set. The diagnostic performance of BreaTB was fairly consistent across different subgroups based on demographic and clinical baseline characteristics, such as age, gender, and anti-tuberculosis therapy. The results demonstrated that age, sex, and anti-tuberculosis therapy have no evident influence on BreaTB. In detail, BreaTB has superior performance on participants with age < 30 than those with age ≥ 30. For different genders, BreaTB also performs slightly differently with superior sensitivity and inferior specificity in females than in males. After the anti-TB therapy, the PTB patients are more difficult to be distinguished from the controls for BreaTB. Except for the general characteristics, we also analyzed the PTB distinguish performance against HC and UHC. BreaTB had a sensitivity of 91.7% (95% CI 87.4–96.0%), and a specificity of 93.5% (95% CI 90.5–96.6%) for the identification of confirmed PTB from HC, which is a quasi-screening scenario. In contrast, inferior specificity of 88.0% (95% CI 75.3–100%) was achieved by BreaTB in distinguishing TB from UHC, which is a quasi-diagnosis scenario.

**Table 4:** Performance metrics (95% CI) of BreaTB on the test data set and on different subgroups

Groups	Sensitivity (%)	Specificity (%)	PPV (%)	NPV (%)	Accuracy (%)	AUC
Overall						
Test (n = 430)	91.7 (87.4–96.0)	93.0 (90.0–96.1)	88.3 (84.5–92.2)	95.1 (91.8–98.4)	92.6 (90.1–95.0)	0.975 (0.961–0.990)
Age, year						
< 30 (n = 233)	90.6 (83.5–97.8)	91.7 (87.6–95.9)	80.6 (74.4–86.7)	96.3 (91.9–100.0)	91.4 (87.8–95.0)	0.972 (0.951–0.993)
≥ 30 (n = 197)	92.5 (87.1–97.8)	95.2 (91.1–99.3)	94.5 (90.2–98.8)	93.4 (88.3–98.5)	93.9 (90.6–97.2)	0.978 (0.958–0.999)
Sex						
Male (n = 243)	90.1 (84.3–95.9)	95.1 (91.5–98.6)	92.9 (88.7–97.0)	93.1 (88.1–98.1)	93.0 (89.8–96.2)	0.975 (0.955–0.994)
Female (n = 187)	94.6 (88.7–100)	90.8 (85.9–95.8)	81.5 (74.7–88.4)	97.5 (93.8–100)	92.0 (88.1–95.9)	0.981 (0.962–1.000)
Anti-TB						
Untreated (n = 385)	92.9 (88.1–97.6)	93.0 (90.0–96.1)	84.6 (80.2–88.9)	96.9 (93.9–100)	93.0 (90.4–95.5)	0.978 (0.964–0.993)
Treated (n = 318)	88.9 (79.7–98.1)	93.0 (90.0–96.1)	67.8 (62.1–73.5)	98.1 (94.6–100)	92.5 (89.5–95.4)	0.968 (0.949–0.987)
Controls						
HC (n = 405)	91.7 (87.4–96.0)	93.5 (90.5–96.6)	90.0 (86.2–93.8)	94.7 (91.2–98.2)	92.8 (90.3–95.4)	0.977 (0.962–0.991)
UHC (n = 182)	91.7 (87.4–96.0)	88.0 (75.3–100.0)	98.0 (93.3–100)	62.9 (55.0–70.7)	91.2 (87.1–95.3)	0.961 (0.933–0.989)

**Fig. 4:**
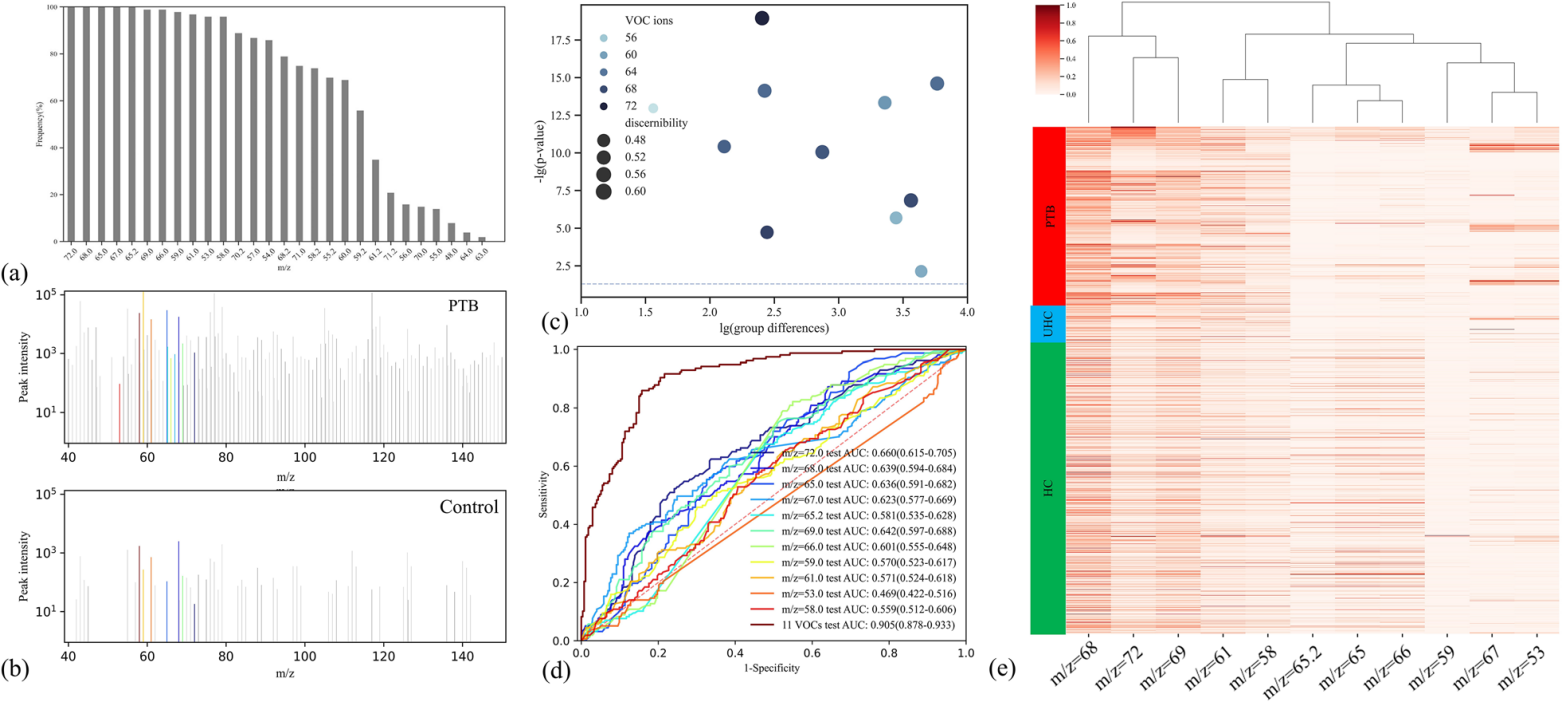
Investigations of breath VOC ions and PTB. **a** The volcano plot shows the group changes and differences in breath VOC ion intensity between PTB and controls. **b** The performances of the top eleven VOC ions in distinguishing PTB patients and controls. **c** The heatmap of the top eleven VOC ions in PTB, UHC, and HC, shows the pattern differences of VOC ions

In this study, over 30 VOC ions were selected via statistical analysis for the BreaTB model training in each iteration. To analyze the importance of different VOC ions for PTB detection, we selected the best VOC ion combinations through RF model based feature selection for 100 iterations. Then, all selected VOC ions were ordered by the selection frequency in RF modeling. As shown in Fig. 4a, there are five VOC ions with m/z of 72, 68, 65, 67, and 65.2 selected at each iteration. There are eleven VOC ions selected in over 90 iterations. Thus, we analyzed the most important eleven VOC ions between confirmed PTB patients and controls. Figure 4b shows the mass spectrum examples of a PTB patient and control individual. It demonstrates that there are some differences in the top eleven VOC ions, which are shown in color bars. To further explore these VOC ions, we analyzed the group differences between PTB and controls and evaluated the performance of each VOC ion in discriminating the PTB and controls. As demonstrated in Fig. 4c, d, all these eleven VOC ions are significantly different between the PTB group and controls with a p-value < 0.05 (the blue line in Fig. 4c). The discernibility (AUC in discriminating PTB group and controls) of VOC ions is related to the scale and significance of group differences. The ROC curve in Fig. 4d shows the discrimination of a single VOC ion is limited (AUC < 0.75). However, the combination of all eleven VOC ions performs well on the test data set with an AUC of 0.905(95% CI: 0.878–0.933). It implies that the panel of VOC ions is the basis for breathomics based PTB detection. The heat map in the PTB group, UHC, and HC illustrated the patterns of these eleven VOC ions are visually different.

**Fig. 5:**
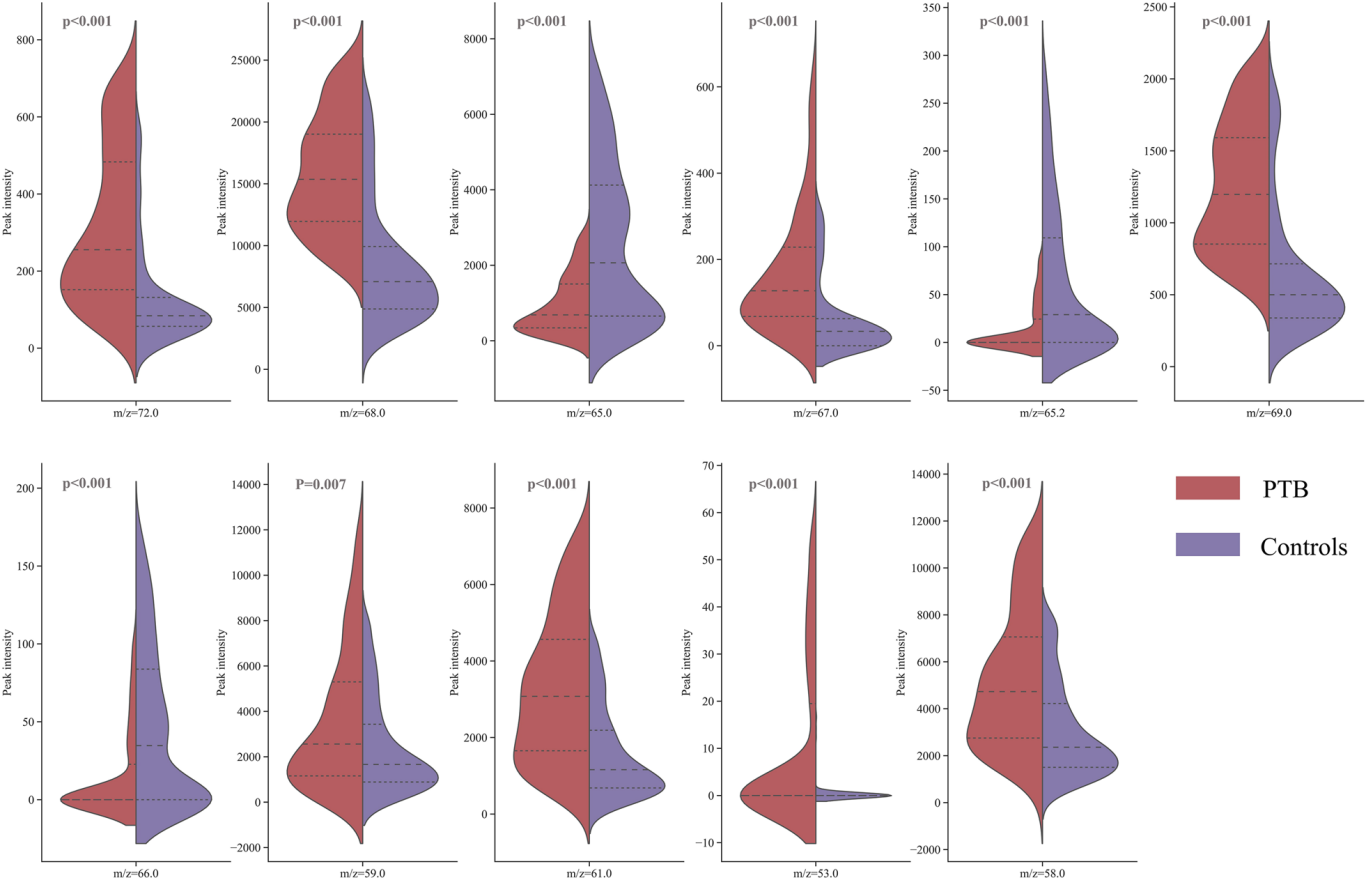
Intensity comparison of VOC ions between PTB group and controls

Since the qualitative ability of the TOF mass spectrometer is limited, we can just infer the possible chemicals of these PTB related VOC ions based on their m/z (72.0, 68.0, 65.0, 67.0, 65.2, 69.0, 66.0, 59.0, 61.0, 53.0, 58.0), correlation-ship (Fig. 4e), intensity distribution (Fig. 5), other published potential biomarkers, and the human breathomics database [39]. Considering the ions intensity distribution similarity and the relationship of m/z values, the VOC ions with m/z of 68 and 69 could be isoprene and its protonated cation. The VOC ions with m/z of 58 and 59 could be acetone and its protonated cation. Isoprene and acetone are common metabolites in human breath [40]. Isoprene is proven to be related to oxidative stress responses [41, 42]. Acetone is related to diabetes [43], and tuberculosis patients have a high incidence of diabetes [44]. The VOC ion with m/z of 72 could be 2-butanone, which is also found as the top eleven biomarkers for PTB in Machel Phillips’s study [17]. The VOC ion with m/z of 61 could be the protonated ions of acetic acid, which was proven related to tuberculosis in skin samples [45]. The VOC ion with m/z of 65, 65.2, and 66 could be the fragment ion of 4-nitrophenol and the corrspounding protonated cation, respectively. The VOC ion with m/z of 67 could be Pyrrole or 3-Butenenitrile. The low peak intensity VOC ions with m/z of 53 could be the fragment ion of other unknown VOC with low concentration. These VOCs would be potential biomarkers of TB.

## Discussion

In this study, for the first time, we explore the diagnostic value of breathomics data detection on HPPI-TOF–MS for PTB in a large cohort. The results demonstrated that the developed BreaTB model performs well in distinguishing PTB individuals and control with high sensitivity and specificity of 91.7% and 93.0%. It implies that the proposed breathomics method via online HPPI-TOF–MS could be a potentially feasible diagnostic or screening tool in the clinical setting.

In the past decades, no breathomics-based method has been translated into clinical practice for the diagnosis of TB, which is primarily due to the complexity and the high cost of existing spectrometers and the limitations of sensor technologies [13]. Compared with past research on PTB detection, there are several advantages in our study. Firstly, the diagnostic accuracy of our VOCs-based PTB detection method was high, with sensitivity and specificity of 91.7% and 93.0%. Furthermore, our study was tested on a large-size patient cohort. As participants were stratified based on their demographic and clinical characteristics: age, sex, and anti-tuberculosis therapy, the diagnostic performance was fairly consistent. Thirdly, TB diagnostic methods using non-sputum samples are strongly advocated by the WHO [46]. Breath sampling has excellent clinical accessibility, especially for certain categories of patients whose sputum is difficult to collect. Fourthly, the breath sample detection on HPPI-TOF-MS only takes about one minute. Thus, the total time cost from breath sampling to getting PTB detection results is about five minutes.

However, there are several limitations in our study. Firstly, the qualitative and metabolic pathways of ions have not been defined. Thus, the logical and mechanistic evidence of the breathomics-based PTB detection method is not enough to make it clinically convincing, although it performs well in clinical data. Further chemical composition analysis via GC–MS is the focus of our future works. Fortunately, many studies have demonstrated the VOCs similarities and differences between the breath of PTB patients and M.tb culture-released gases. For example, Phillips et al. found the common compounds: 1-methyl- and cyclohexane, 1,4-dimethyl- in the breath of PTB patients and headspace air of culture [17]. Using computational approaches, Purva et al. proposed putative biosynthetic pathways in M.tb for three VOCs(methyl nicotinate, methyl phenylacetate, and methyl p-anisate), and methyl nicotinate was also found in the exhaled breath of patients with tuberculosis [47]. Kuntzel et al. detected and analyzed the headspace VOCs of 17 different mycobacteria and control strains. Their result demonstrated the feasibility of identifying M.tb from other pathogens based on their metabolism of VOC [48]. Our team is also working on finding the links between the VOCs in the breath samples of PTB patients and the VOCs in the headspace air of M.tb culture. Secondly, the control group contained only a small sample of patients with pulmonary diseases other than PTB. Thus, the performance needs to be further evaluated in detecting PTB from other pulmonary diseases. Thirdly, our enrollment was restricted to adults with possible PTB. Similar independent validation studies are needed for children whose diagnostic tools are even more urgently needed [21], as well as patients living with diabetes or HIV and patients suspected of EPTB. At last, this is a single-center study conducted in a TB specialist hospital, which may limit the universality of the research results.

## Conclusion

In conclusion, we developed a breathomics model: BreaTB for PTB detection, which achieved high diagnostic accuracy on clinical data set with a sensitivity and a specificity of 91.7% and 93.0%, respectively. Due to its simplicity and low cost, the breathomics-based PTB detection model on online breath analysis platforms such as HPPI-TOF-MS has the potential to meet the ongoing demand for TB diagnosis that would not require sputum and may work in active case finding in large populations, especially in resource-limited settings where it is urgently needed [12]. However, more clinical and basic researches are needed to evaluate this method in patients with more complex health conditions and with various lung diseases. Last but not least, more studies are needed to confirm the TB-specific breath biomarkers and clarify their metabolic pathways.

## Data Availability

The datasets used or analysed during the current study are available from the corresponding author on reasonable request.
